# Efficacy of multisystemic therapy in youths aged 10–17 with severe antisocial behaviour and emotional disorders: systematic review

**DOI:** 10.1080/17571472.2017.1362713

**Published:** 2017-08-09

**Authors:** Jia Xuan Tan, Maria Lourdes Restrepo Fajardo

**Affiliations:** Department of Child and Adolescent Psychiatry, Institute of Psychiatry, King’s College London, London, UK

**Keywords:** Multisystemic therapy, antisocial behaviour, youths, delinquency, emotional disorder

## Abstract

**Background:**

Antisocial behaviour and conduct disorders are the most common behavioural and mental health problems in children and young people globally. An efficacious intervention is needed to manage these antisocial behaviours that have costly consequences. Multisystemic Therapy (MST), an intensive home-based intervention for youths with psychosocial and behavioural problems, is recommended under National Institute for Health and Clinical Excellence guidelines for conduct disorder. However, reviews on the efficacy of MST are mixed.

**Aim:**

To review randomised controlled trials (RCTs) reporting efficacy of MST among youths presenting with antisocial behaviour and emotional disorder respectively.

**Method:**

A systematic map term to subject heading search was conducted in PsycINFO, Embase, and Ovid Medline databases for articles up to November 2015. RCTs comparing MST vs.treatment as usual (TAU) in youths presenting with antisocial behaviour and emotional disorder were included.

**Results:**

12 RCTs (*n* = 1425) reported efficacy of MST vs. TAU in youths presenting with antisocial behaviour and emotional disorder. Clinically significant treatment effects of MST showed a reduction of antisocial behaviour which includes delinquency. MST, vs. psychiatric hospitalisation, was associated with a reduction of suicidal attempts in youths presenting with psychiatric emergencies. 4 studies showed that MST was less costly than TAU in the short term, with further analysis required for long-term cost-effectiveness.

**Conclusion:**

MST is an efficacious intervention for severe antisocial behaviours in reduction of delinquency and should be included in clinical practices. MST was shown to have a positive effect on emotional disorder but further research is needed to evaluate the efficacy of MST with emotional disorder. Further analysis is required to assess the services utilized for long-term cost effectiveness.

## Key messages

• The commonest child and adolescent psychiatric disorder, conduct disorder, involves antisocial behaviour.
• Long-term financial cost to the public of antisocial behaviour is immense, thus having primary care involved in prevention and treatment is important.
• Multisystemic therapy (MST) is an efficacious intervention for severe antisocial behaviour in reduction of delinquency.
• Primary care practitioners could facilitate treatment by referring youths with severe antisocial behaviours which greatly impact their daily functioning to mental health professionals for MST.

## Why this matters to me?

The long-term negative impact of the effects of antisocial behaviour is serious, as it not only affects the individual, but also affects the family and the entire society. Persistent antisocial behaviour leads to increased risk of criminality, unstable relationships and mental health problems. Primary care practitioners have a particularly important role as the first line of service provider in prevention and identification of antisocial behaviour, and referring efficacious intervention of MST for severe antisocial behaviour.

## Introduction

### Background

Antisocial behaviour which includes delinquency during childhood and adolescence is common. It can have significant and costly long-term consequences for individuals, families, and society [1,2]. Peer rejection and school drop-out are usually associated with aggressive and disruptive behaviour among younger children [3]. The most antisocial 5% of 7-year old children are 5 to 10 times more likely to result in failures in life [4]. There is an urgent need for effective intervention to manage antisocial behaviour which leads to costly consequences [5].

### Multisystemic therapy

MST is an intensive family and community-based intervention for youths with severe psychosocial and antisocial behavioural problems [6]. It was developed to target the multi-determined nature of antisocial behaviour and addresses all environmental systems that impact juvenile offenders [6]. To ensure a high level of treatment fidelity, intensive training, supervision and weekly integrity checks are conducted by an expert in MST [7]. Research has also shown that MST can be considered as an alternative to inpatient care [8].

The target population has expanded from juveniles with antisocial behaviour to those with sex offence convictions [9,10], emotional disorder [11,12], history of substance abusing [13] and chronic physical illness [14]. It is currently applied widely in the United States[7,9–13,15–17] and also in Norway [18], Sweden[19] and the United Kingdom [20].

### Previous reviews

A recent guideline for management of antisocial and conduct disorder in children and young people under the National Institute for Health and Clinical Excellence (NICE) [5] suggests MST as one of the multimodal interventions. Positive reviews on efficacy of MST, indicated by 2 meta-analyses, showed that youth and families receiving MST function better than 70% of the alternative group [21]. It has the most effect with sex offenders and larger effects when compared to a non-multimodal treatment [22]. However, a systematic review of MST for emotional and behavioural problems in youths aged 10–17 found inconclusive evidence of the effectiveness of MST compared with other interventions [23]. The efficacy of MST from previous reviews remains controversial [21–23], and an updated review of the efficacy of MST is required.

### Scope

This review seeks to extend and update previous reviews [21–23] focusing on randomized controlled trials (RCTs) of MST vs. TAU in reducing antisocial behaviour and emotional disorder among youths.

## Methods

### Search strategy

A systematic map term to subject heading search was conducted in PsycINFO, Embase, and Ovid Medline databases for articles up to November 2015. Keywords used were multisystemic therapy* AND mental health* OR mental disease* OR mental health service* OR mental disorder*(Appendix 1). Reference lists of articles were examined, and an expert in the field was contacted for relevant articles. Two reviewers (JT and MLR) screened for all abstracts independently and no disagreements identified. Each study was assessed for allocation concealment [23,24]. Jadad score was calculated with a maximum score of 5 for each of the included RCTs. The Jadad score reflects the quality of the study, such as the quality of randomization, blinding procedures, and description of withdrawals and dropouts [25].

#### Inclusion criteria

RCTs with MST as the intervention group and control as the treatment-as-usual (TAU) group and with study population of children or adolescents aged 10–17 with behavioural and emotional problems were included.

#### Exclusion criteria

Studies that did not conform to current criteria for evaluating methodological quality of RCTs (Jadad score <2), not published in English and focus on chronic physical conditions exclusively. Review papers, ongoing trials, follow-up studies and studies which did not examine intervention efficacy were excluded as well.

## Results

### Data extraction

146 articles were identified from the database search. 2 hand-searched articles were identified through reference lists of relevant articles and 2 articles from an expert in the field. 43 articles were identified as duplicates. In total, 107 abstracts were reviewed and 38 articles qualified for full-text screening.

**Figure 1.**
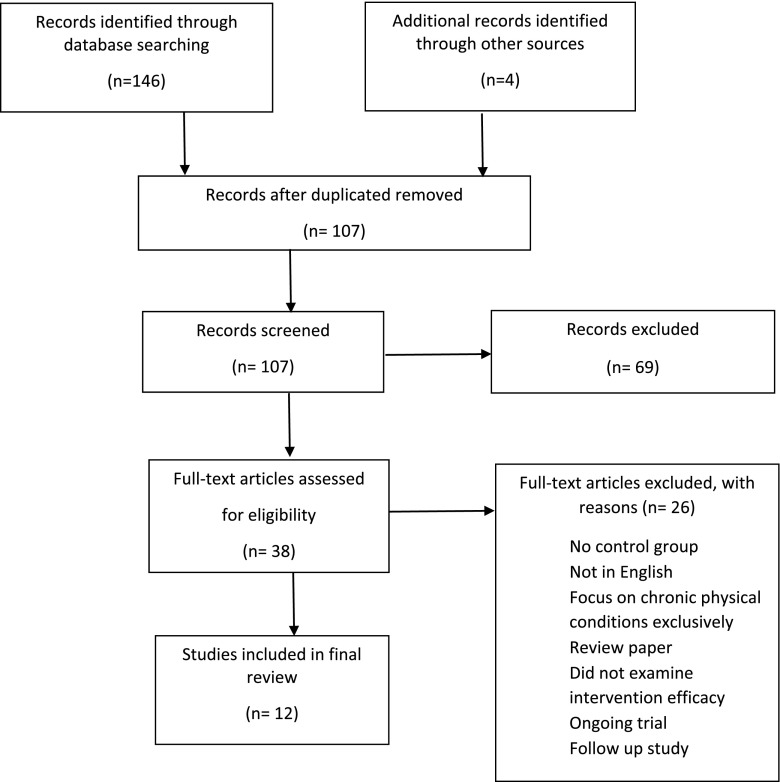
Study flow.

After full-text examination, 12 articles (*n* = 1425) which fulfilled the full inclusion criteria were included. The study flow chart is provided in Figure 1. Study characteristics have been summarized in Table 1.

**Table 1.** Characteristics of included studies

Authors (Year) Country	Population	Age	*N*	Treatment	Duration	Main measures	Main findings (Delinquency and incarceration)
*Multisystemic therapy with serious juvenile offenders*							
Henggeler et al. (1992) USA	Violent and chronic juvenile offenders and their families	11–17	84	(1) MST (2) Court order with different stipulation	T1: Baseline	Arrest and incarceration period; CBCL; SRD; FACES-III; MPRI; RBPC; SCL-90-R	• 80% (vs 32%) of MST youths were not incarcerated (*d* = .62; *p* < .006) and 58% (vs 38%) of youths experienced no arrests (*d* = .45; *p* < .05) • Increased family cohesion and decreased youth aggression in peer relations
					T2: 59 wks		*Treatment fidelity*: Treatment and supervision were less extensive but findings reflect successful diffusion of MST in community setting
Henggeler et al. (1997) USA	Violent and chronic juvenile offenders and their families	11–17	155	(1) MST (2) Probation and other social service agencies (TAU)	T1: Baseline	Arrest and incarceration history; SRD; FACES-III; MPRI; GSI; RBPC; FAM-III; Monitoring index	• 26% reduction in re-arrest (not clinically significant), 47% reduction in days incarcerated, *F* (1,151) = 7.26, *p* < .008, for youths in the MST group from T1 to T3.
					T2: Post-treatment		*Treatment Fidelity*: Better outcomes with high treatment fidelity. Importance of maintaining treatment fidelity in disseminating services to the community
					T3: 1.7 yrs		*Cost effectiveness*: MST is more cost effective than TAU by reduction rate of incarceration
Henggeler et al. (1999) USA	Substance-abusing and -dependent delinquents and their families	12–17	118	(1) MST (2) Outpt substance abuse services	T1: Baseline	Arrest and incarcerated records; SRD; PEI; Addiction severity index; Youth risk behaviour survey	• Drug use (T1 to T2) of youth reports on alcohol/ marijuana use *F*, (1, 112) = 5.40, *p* < .022 • Significant decreases in self-reported offending for time in between time periods T1–T2, T1–T3, T2–T3, *t*^HSD^ = 7.72, 11,46, 3.87 respectively, *p* < .05 • No statistically significant results for arrests (26%) and recidivism (19%)
					T2: Post treatment		
					T3: 6 mths		
					T4: 12 mths		*Treatment fidelity*: Treatment fidelity was low, associated with modest results of study
					T5: 4 years		
Timmons-Mitchell et al. (2006) USA	Juvenile offenders at risk of placement and their families	Mean age 15.1	93	(1) MST (2) TAU	T1: Baseline	Arrest and incarceration history; Child and adolescent functional assessment scale (CAFAS)	First RCT without involvement of MST developers
					T2: At discharge		• Significant reduction in re-arrests. MST (66.7%) and TAU (86.7%), *X*^2^ (1) = 5.14, *p* < .05. • MST reported fewer new offenses (*M* = 1.44, SD = 1.5) than TAU (*M* = 2.29, SD = 1.5) (*p* < .01) • Improvement in 4 areas of functioning measured by the CAFAS for youths who received MST
					T3: 6 mths		
					T4: 12 mths		*Treatment Fidelity*: High TAM ratings across all therapists was 4.2; Similar to a larger study examining transportability of MST
Letourneau et al. (2009) USA	Juvenile sexual offenders and their families	11–17	127	(1) MST (2) TAU – Juvenile sex offender (TAU-JSO)	T1: Baseline	Arrest and incarceration history; CBCL; SRD; PEI; Adolescent sexual behaviour inventory (ASBI)	• Significant negative linear effects for reduction in problem sexual behaviour, self-reported delinquent behaviour and substance use (*p* < .001) for MST group • Community-based and family-focused interventions were more effective than TAU-JSO
					T2: 6 mths		*Treatment fidelity*: High level of treatment fidelity with weekly supervision and on-site MST supervisor
					T3: 12 mths		*Cost effectiveness*: MST is more cost effective than TAU- JSO due to prevention of crime and reduction of out-of-home placements
Borduin et al. (2009) USA	Juvenile sexual offenders and their families	Mean age 14	48	(1) MST (2) Cognitive-behavioural therapy and individual treatment through juvenile court	T1: Baseline	Arrest and incarceration history; SRD; GSI; RBPC; FACES-II; MPRI; School grades	• MST had 70% fewer arrests for all crimes and spent 80% fewer days confined in facilities than TAU • MST participants had lower risk for sexual offenses, *x*^2^(1, *N* = 48) = 11.80, *p* < .001 and nonsexual crimes, *x*^*2*^ (1, *N* = 48) = 3.94, *p* < .05.
					T2: Average 8.9 years after treatment has been completed		*Treatment Fidelity*: High treatment adherence by direct supervision of author. Contributed to a comprehensive intervention and positive results
							*Cost-effectiveness*: MST is more cost effective than TAU
Butler et al. (2011) U.K.	British juvenile offenders and their families	13–17	108	(1) MST (2) Individual treatment by Youth offending team (YOT)	T1: Baseline	Arrest and incarceration history; CBCL; SRYB; Antisocial beliefs and attitudes scale (ABAS)	• Significant reduction in number of offenses at T2 • Rates of non-violent offending reduced significantl*y* (*z* = 3.29, *p* < .001) during the 18-month follow up for MST group • Youth-reported delinquency, parental-reported aggressive and delinquent behaviour showed significantly greater reductions from pre-treatment to post-treatment levels in the MST group
					T2: 6 mths		*Treatment fidelity*: No significant effect of TAM scores to the primary outcome variables
					T3: 18 mths		
*Multisystemic therapy with youths with severe conduct problems*							
Ogden et al. (2004) Norway	Norwegian youth with serious antisocial behaviour and their families	Ave age 14.95	100	(1) MST (2) Usual child welfare services	T1: Baseline	Arrests and incarceration history; CBCL; SRD; SCPQ; SSRS; FACES-III	• MST: Significant decreased externalising (*F*_1,88_ = 3.34, *p* = .07) and internalising symptoms (*F*_1,86=_4.67, *p* = .03) • MST: Significant decreased out-of-home placements (90.6% MST youths at home vs 58.1% TAU youths at home at T2)
					T2: 6 mths		*Treatment fidelity*: TAM scores differed significantly across 3 sites, site with the lowest score resulted in least favourable outcomes and vice versa
Sundell et al. (2008) Sweden	Swedish youth with conduct disorder and their families	12–17	156	(1) MST (2) TAU	T1: Baseline	CBCL; SRD; SCPQ; SSRS; SCL-90-R; School attendance; Alcohol and drug consumption	• No significant difference in treatment effects between the MST and TAU groups with both groups improved.
					T2: 7 mths		*Treatment fidelity*: TAM significantly correlated with 2 of the outcome measures (Arrest; Social competence with peers)
Weiss et al. (2013) USA	Adolescents with serious conduct problems in self-contained classrooms and their families	11–18	164	(1) MST (2) TAU	T1: Baseline	Arrests history; CBCL; SRD; FACES-III; School grades; School attendance	• MST parents and adolescents reported significantly greater rates of decrease in externalising problems. • No significant differences on delinquent behaviour or drug use and arrests
					T2: 3 mths		*Treatment Fidelity*: Sessions were audiotaped for fidelity coding and moderately high to high therapists’ adherence were recorded
					T3: 6 mths		
					T4: 18 mths		
*Multisystemic therapy with emotional disorder*							
Henggeler et al. (1999) USA	Youth presenting psychiatric emergencies and their families	11–17	116		T1: Baseline	CBCL; PEI; GSI; FFS; FACES-III; School attendance	More effective than inpatient psychiatric hospitalisation
				(1) MST (2) Inpt			• Significant decrease in externalising symptoms (*F*_2204_ = 3.99, *p* < .021) at T3 as compared to inpatient (inpt) psychiatric care. • Significant improvement in family adaptability (*F*_2220_ = 3.48, *p* < 0.39) and cohesion (*F*_2206_ = 6.56, *p* < .001)
					T2: 1–2 wks		*Treatment fidelity*: Intensive supervision from psychiatrist. Fidelity ensured through structured MST supervision and coded audiotapes
				Psychiatric care	T3: 4 mths		
Huey et al. (2004) USA	Referred for emergency psychiatric hospitalisation	Ave age 12.9	156		T1: Baseline		1st study of MST for suicidal behaviour in children and adolescents. More effective than inpatient psychiatric hospitalisation:
				(1) MST (2) Inpt			• Significant decrease in rates of attempted suicide at 1-year follow-up (*p* < .01) • Significant decrease in rate of symptom over time • (*p* < .001) • No long-term differential effects on suicidal ideation, youth depressive affect or youth-rated parental control.
					T2: 4 mths		*Treatment fidelity*: Adapted MST treatment principles for the treatment of youths in psychiatric crisis. Particular attention was given to targeting the methods used by the youth in previous suicidal episodes
				Psychiatric care	T3: 1 year	CBCL; FFS; GSI; Hopelessness Scale for Children; Youth risk behaviour survey	*Cost effectiveness*: Further analysis done. MST has better short-term cost effectiveness for each of the clinical outcomes (externalising behaviour, internalising behaviour and global severity of symptoms) than inpatient and community care. Equivalent long-term cost-effectiveness

### Efficacy of MST with youths with severe antisocial behaviours (Serious juvenile offenders)

All 7 studies showed clinically significant treatment effects of the MST group as compared to the TAU group. The 2 main treatment outcomes of MST were found to be reduction in incarceration and delinquency; defined as any illegal activity [22]. The group receiving MST showed a reduction in delinquency, incarceration [9,10,15,16,20] and sex-offending behaviour [9,10] (Refer to Table 1). However, 2 other studies showed insignificant treatment effects in reduction of rearrests [7,13]. This is further supported by an independent replication of the study conducted in the United States [16] demonstrating significant treatment outcomes of MST in the reduction of delinquency.

### Efficacy of MST with youths with conduct disorder

2 out of 3 studies showed positive outcomes of MST in reduction of antisocial behaviour (i.e. aggressive and non-compliant) [17,18] and emotional problems (i.e. anxious and depressive) [18] (Refer to Table 1). As the study was conducted across 4 sites [18], the treatment fidelity varied significantly with low treatment fidelity scores associated with least favourable outcomes and vice versa.

In contrast, another study in Sweden [19] reported no significant difference in treatment effects between the MST and the TAU group. Although the fidelity to the treatment was lower than other studies, there is no clear association with the negative outcomes of this study.

### Efficacy of MST with youths with emotional disorders

In terms of suicidal youths presenting with psychiatric emergencies, both studies [11,12] reported significant treatment effect of MST. Henggeler et al. [11] reported significant differences in the treatment effect of MST for youth antisocial behaviour, especially at long term follow-up as compared to the control group. Similarly for the study on suicide attempts by youths [12], treatment effects of MST were significant in reduction of attempted suicide at 1 year follow up. No treatment effects on suicidal ideation, youth depressive affect or youth-rated parental control were reported in the long-term.

### Cost

Four studies explored the cost effectiveness of MST vs. TAU. The cost savings of MST were based on the prevention of crime [10] and reduced incarceration per year [7,9]. MST was less costly than usual services in the short-term, but no analysis was conducted for long-term cost effectiveness. An assessment of service utilization across service sectors (e.g. mental health, juvenile justice, social welfare) is needed to fully explicate the types of services received by the youths to explore if costs shifted in the long-term [7,9].

## Discussion

Firstly, MST is efficacious for youths with severe antisocial behaviour with treatment adherence as a predictor for key outcomes of delinquency and incarceration. Secondly, it appears to be effective for youths with severe conduct disorders from a non-court system and youths with emotional problems.

### Youths with severe antisocial behaviour (serious juvenile offenders)

MST is efficacious for youths with severe antisocial behaviour including sexual offenders, as it targets the determinants of antisocial behaviour and perpetuating factors of sexual behaviour [9,10] in the natural environment with youths and families [7]. The reliability of the positive results is supported by multiple methods of measurement and by data collected in the long-term (59 weeks post-referral) [26].

5 articles [7,9,10,13,16] with juvenile offenders found the association of treatment adherence to outcomes. Poor outcomes were associated with low emphasis of the therapist changing family interactions and lack of direction in therapy [7]. In addition, Borduin et al. ensured a high treatment fidelity through direct supervision. This has thus contributed to a comprehensive intervention which yielded efficacious results in reducing risks for sexual and nonsexual criminal activity in juvenile offenders [9].

In contrast, a study [15] conducted by community mental health professionals showed successful implementation of MST in the community setting, with a less extensive supervision basis [27,28]. Although there were two studies [15,20] which showed no association between treatment adherence and outcomes, the majority of the studies proposed that treatment adherence is an important predictor for key outcomes of delinquency and incarceration [6].

### Youths with severe conduct disorder

MST appears to be less efficacious in reduction of less severe antisocial behaviour (delinquency, drug use, and arrests rates) [17,18]. Possible reasons are firstly, MST is more suitable for more serious antisocial behaviour in juvenile offenders as compared to youths with conduct disorder.

Secondly, treatment fidelity could possibly account for the modest results obtained. Low fidelity scores could point to lack of robust implementation of MST in the Swedish study which resulted in insignificant difference in treatment effects [19]. With treatment not mandated by the court, the motivation of parents and individuals could also be affected.

Thirdly, multimodal services provided to the control group could be another possible explanation, which is in line with the findings of meta-analysis of effectiveness of MST in van der Stouwe et al. [22] Offenders in Sweden and Norway are referred to the social services as there are no legal sanctions imposed on them [19]. Thus, a broad array of social services and mental health treatments [18], which are similar to MST, are provided to youths through a child welfare approach.

### Youths with emotional disorder

In comparison with psychiatric hospitalisation, MST was shown to decrease emotional disorder and improve on the family system of the individual [11]. Huey et al. [12] conducted the first study to explore the effects on attempted suicide by youths. Although significant results of the reduction of attempted suicide was reported one year post-treatment with MST intervention, the three most robust predictors of attempted suicide (depressive affect, hopelessness and suicidal ideation) did not show any treatment effects [12]. However, literature has also shown that interventions which are successful in treating the predictors mentioned have shown minimal effects in reducing the behaviour of attempted suicide [29,30], thus it is important not to overly state the correlation of suicidal risks with feelings of hopelessness and depressive affect. With limited evidence, possible key factors gathered from two studies suggest access to hospitalisation for crisis management before MST is implemented and a coordinated care plan are important [11].

Another important factor to consider is self-harm, a strong predictor of eventual death by suicide [31]. The Self Harm Questionnaire (SHQ) can be used in primary care for early identification of self-harming adolescents that warrant a secondary care referral and to facilitate early intervention. It aims to identify self-harm thoughts and behaviour in psychiatrically referred adolescents and also a allow a detailed assessment of the most recent episode of self-harm [32].

### Comparison with other reviews

Findings of this review are in line with the meta-analysis [21] which showed the efficacy of MST in treating antisocial behaviour, with reduction in delinquency and better functioning families. The results are aligned with NICE guidelines [5] which suggested the use of multimodal interventions. Larger effects of MST when compared with a non-multimodal treatment [22] are also confirmed. Functional Family Therapy (FTT) is another example of a multimodal intervention which is a family-based intervention programme for youth with behavioural problems [33]. In line with other reviews’ recommendations, more research in the efficacy of MST with emotional disorders is needed.

## Limitations

First, all studies included were implemented in United States or in European countries, thus results may not be applicable to other culturally different countries. Second, the TAM tool is not unique to MST and constructs such as engagement and therapeutic alliance are measured, which makes discriminating between the various constructs associated with the treatment outcomes difficult. Furthermore, there is no standardized protocol in measuring treatment fidelity. Third, the allocation concealment was evident in only 6 included studies and thus, the possibility of selection bias in assigning participants to their given treatment could not be ruled out. Fourth, possibility of retrieval bias as only English language journals were selected. Lastly there are limited studies available for populations other than serious juvenile offenders, thus there is insufficient evidence to conclude the efficacy of MST for youths with emotional disorders.

## Conclusion

MST is an efficacious intervention for severe antisocial behaviour in reduction of delinquency and incarceration thus it should be recommended for clinical practice. It is shown to have a positive effect on emotional disorder but further research is needed. Treatment fidelity is a crucial consideration factor to ensure high efficacy. Further research is needed to address the cultural relevance of MST to the UK. For countries with well-developed and comprehensive social services for youth delinquents, more research is needed to evaluate efficacy of multimodal treatment approaches (e.g. MST).

## Disclosure statement

No potential conflict of interest was reported by authors.

## Governance information

Kings College London, Department of Child and Adolescent Psychiatry, Institute of Psychiatry oversaw this work.

## Appendix 1. Map term to subject heading search.

**Table:**

	Searches	Results
*Database 1: PsycINFO* 1806–November Week 3 2015		
# 1	Exp mental health services/ or exp mental health/ or exp mental disorders/	542788
# 2	Exp multisystemic therapy/	156
# 3	1 and 2	29
*Database 2: Embase* 1974–2015 Nov 20		
# 1	Multisystemic therapy*.mp.	199
# 2	Exp mental health service/ or exp mental health/ or exp mental disease/	1782992
# 3	1 and 2	115
# 4	Limit 3 to (randomized controlled trial and (school child < 7–12 years > or adolescent < 13–17 years>))	18
*Database 3: Ovid medline* **®** 1946 to November 2 2015		
# 1	Multisystemic therapy*.mp.	147
# 2	Exp mental health services/ or exp mental disorders/ or mental health*.mp.	1113265
# 3	1 and 2	103
# 4	Limit 3 to ‘all child (0–18 years)’	**99**
	Total No. of articles	146
